# Surface Defect Segmentation Algorithm of Steel Plate Based on Geometric Median Filter Pruning

**DOI:** 10.3389/fbioe.2022.945248

**Published:** 2022-07-01

**Authors:** Zhiqiang Hao, Zhigang Wang, Dongxu Bai, Xiliang Tong

**Affiliations:** ^1^ Key Laboratory of Metallurgical Equipment and Control Technology of Ministry of Education, Wuhan University of Science and Technology, Wuhan, China; ^2^ Hubei Key Laboratory of Mechanical Transmission and Manufacturing Engineering, Wuhan University of Science and Technology, Wuhan, China; ^3^ Precision Manufacturing Research Institute, Wuhan University of Science and Technology, Wuhan, China; ^4^ Research Center for Biomimetic Robot and Intelligent Measurement and Control, Wuhan University of Science and Technology, Wuhan, China

**Keywords:** structured pruning, model compression, semantic segmentation, defect detection, deep learning

## Abstract

Problems such as redundancy of detection model parameters make it difficult to apply to factory embedded device applications. This paper focuses on the analysis of different existing deep learning model compression algorithms and proposes a model pruning algorithm based on geometric median filtering for structured pruning and compression of defect segmentation detection networks on the basis of structured pruning. Through experimental comparisons and optimizations, the proposed optimization algorithm can greatly reduce the network parameters and computational effort to achieve effective pruning of the defect detection algorithm for steel plate surfaces.

## 1 Introduction

Applying defect detection segmentation algorithms to real industrial production scenarios, hardware resources are a challenge that must be faced (Tang et al., 2017; Liu H. et al., 2020; Hao et al., 2021). Complex models often mean better detection capabilities, but the high memory space footprint and huge consumption of computational resources doom it to ineffective application in resource-limited hardware platforms (Sun H. et al., 2020, Sun et al., 2022c; Tao et al., 2022; Wu et al., 2022; Zhao et al., 2022). Therefore, compression of redundant neural network models is essential.

Model pruning is a fast and effective way to compress neural networks by cutting out unimportant neurons or filters to obtain a small network model with small storage capacity and fast inference. Model pruning can inherit the weights of the network before pruning, so the model can be pruned to achieve better optimization results.

Model pruning is a fast and effective way to compress neural networks by cutting out unimportant neurons or filters to obtain a network model with small storage capacity and fast inference. Model pruning inherits the weights of the network before pruning, so model pruning allows for better mobile deployment and better optimization.

For real-time applications such as surface EMG signal processing (Li et al., 2019b, Li et al., 2020; Sun et al., 2020a; Qi et al., 2020; Yang et al., 2021), gesture recognition (Duan et al., 2021, Liu X. et al., 2022, Liu et al., 2022a, Liu et al., 2022b, Luo et al., 2020, Jiang et al., 2019a, b, Xu et al., 2022, Sun et al., 2022a) and quality inspection (Chen et al., 2021a, Chen et al., 2021b, Huang L. et al., 2021, Jiang et al., 2021a, Jiang et al., 2021b, Sun et al., 2021b, Chen et al., 2022a, Chen et al., 2022b, Chen et al., 2022c, Huang et al., 2022, Sun et al., 2022b, Yun et al., 2022b, Zhang et al., 2022), model compression effectively reduces the memory and computational power consumed by the original large neural network, and improves the training and inference speed. Moreover, the compressed models are conducive to deployment and timely updates on embedded and mobile devices with limited storage space, facilitating the development of smart factories (Li et al., 2019a; Li et al., 2019c; Yun et al., 2022a).

The key contributions of this work are:

1) A model pruning method based on improved geometric median filter pruning is proposed on the basis of structured pruning.
2) The pruning method and pruning process are improved by performing model acceleration and fine-tuning in the structured pruning process, and determining whether the pruning end condition is satisfied by the evaluation function to improve the pruning compression efficiency.
3) After experimental comparison, the improved geometric median filter model-based pruning method proposed in this paper outperforms other classical pruning methods. And the pruning algorithm has better detection performance and pruning efficiency in steel plate surface defect segmentation detection.

The rest of this paper is organized as follows: Section 2 discusses related work on model compression in recent years. Section 3 analyses model pruning methods and clarifies in detail the advantages and disadvantages of unstructured pruning and structured pruning methods; and as a basis, proposes a structured model pruning method based on geometric median filtering for pruning and compressing steel plate surface defect models. After a brief introduction of the open source steel plate surface defect dataset and the configuration of the experimental environment, Section 4 presents an experimental comparison of the proposed pruning algorithm with other pruning algorithms to demonstrate the effectiveness of the structured pruning algorithm. Section 5 concludes the paper with a prospect.

## 2 Related Work

In recent years, in order to perform more complex information processing tasks, deep learning-based neural network models have become deeper and deeper, also making them increasingly computationally intensive, making it difficult to deploy neural networks on devices with scarce computational resources or with strict latency requirements (Liu H. et al., 2022). As a result, compression of neural network models is becoming increasingly important. For applications such as steel plate surface defect detection, where real-time requirements are particularly stringent, it is even more important to reduce the computational cost and storage requirements and to speed up the computation. Currently, there are five main neural network model compression methods that are widely used (Gao et al., 2021): Low rank decomposition, structural design, knowledge distillation, parameter quantization and model pruning, and the relevant short descriptions are shown in Table 1.

**TABLE 1:** Model compression methods.

Methods	Method description	Advantages and disadvantages
Low-rank decomposition	Low-rank decomposition of parameter matrices	Parameter matrix decomposition is more difficult and requires larger hardware resources
Structural design	Designing special convolution kernels	Constructing new modules, trained from 0
Knowledge distillation	Train to optimise your network with a large model as a guide	Training from 0, model performance is more sensitive to network structure is more sensitive
Parameter quantification	Replacing high-precision weighting parameters with low precision	The quantified parameters are often not derivable and the actual update may deviate from the original gradient direction
Model pruning	Crop parameters that are not important to the final accuracy	The pruned model has some robustness and can achieve better optimization

Liu M. et al. (2020) proposed a joint optimization model of low-rank matrix bi-factor decomposition and structured sparse matrix decomposition, and applied it to saliency target detection with low time complexity. Zhang and Chen (2019) modelled the detection of defects on the track surface as a low-rank matrix decomposition problem, and calculated the row accumulation of the sparse matrix obtained from the decomposition, and searched for the maximum connected region to determine the defect location, realizing automatic detection and localization of defects. Wang et al. (2018) used multiple independent and complementary information in the multi-view feature space to outperform single information, and proposed that by decomposing the potential low-dimensional data cluster representations to present structured low-rank representations and improve clustering performance by exploring multi-view consensus structures beyond low-rank with an efficient alternating minimization strategy function. Ouyang (2021) proposed an improved autoencoder architecture based on an extreme learning machine that uses low-rank matrix decomposition to learn optimal low-dimensional features. The representational and non-linear capabilities of the features are enhanced. However, due to the large arithmetic size of matrix decomposition, it inherently takes longer training time and requires more hardware resources.

DenseNet (Huang et al., 2017) is a densely connected neural network, with connections between any two layers of the network, combining information from all previous layers as input features for the next layer and introducing a feature channel scaling factor and a resolution scaling factor into the network, further reducing the computational effort of the network. Inception (Szegedy et al., 2016), on the other hand, uses mainly 1 × 1 filters instead of 3 × 3 filters, saving the number of parameters in the network. To randomly disrupt the feature channels, ShuffleNet (Xin et al., 2021) divides the feature channels into multiple groups and convolves them to increase the information exchange between different feature channels. MobileNet (Sun et al., 2021a) designs a deeply separable convolution module and fuses the information of different feature channels by 1 × 1 convolution. In addition, researchers often introduce 1 × 1 filters between 3 × 3 filters to reduce the number of input and output channels of the feature map. Although lightweight networks are effective in reducing the computational complexity of the network, there is still a large amount of redundancy in the network and the design requirements are high.

Huang J. et al. (2021) replaced the traditional static convolution by constructing a dynamic convolution module incorporating an attention mechanism to transfer dynamic feature knowledge from the teacher network back to the student network, thus achieving high accuracy recognition of defects while significantly reducing model inference time. Liu et al. (2021) proposed a neural network compression algorithm based on knowledge distillation and adversarial learning, and allowed the teacher network and student network to learn from each other in the second half of training, enabling the student network to explore its own optimal solution space. Park and Yong (2020) proposed to apply channel and spatial correlation loss functions and adaptive cross-entropy loss functions to train the light network and use the heavy network for semantic segmentation. Knowledge distillation from the heavy network as the teacher to the light network as the student can be used as a way to improve the performance of the student network. Zhang et al. (2021) proposed a novel two-branch network that took three pairs of original transformed images as input and incorporated a class activation graph to drive the network to mine the most relevant class-specific regions. This strategy ensured that the network generated differentiated embeddings and a round of self-knowledge distillation was set up to prevent overfitting and improve performance. However, compared to other compression methods (Sarakon et al., 2021), the whole training process of knowledge distillation takes longer and is only applicable to neural networks with softmax layers.

Rao et al. (2019) proposed a deep neural network compression method based on dynamic quantization coding, in which the quantization codebook is updated simultaneously during the training of the model, so that the codebook minimizes the error caused by quantization of larger weight parameters. Sun H. et al. (2020) proposed a lightweight image compression neural network based on parameter quantization, quantizing the model parameters from 32-bit floating-point to 8-bit integer, saving 73% of storage space compared to the original model. Chen et al. (2019) proposed an efficient convolutional neural network-based fast decision method for quantization parameter selection for video coding by comparing the rate distortion cost to calculate the optimal quantization parameters, saving the encoding time of the video. Feature extraction is important for steganalysis of content-adaptive JPEG steganography, Xu et al. (2018) proposed a scale covariance matrix feature based on a two-dimensional Gabor filter and used diverse quantization of filter residuals to improve detection performance.

Jin et al. (2018) proposed a hybrid pruning method combining weight pruning and convolutional kernel pruning; the convolutional kernels that contribute less to the overall accuracy of the convolutional neural network are pruned first, and then the pruned model is weight pruned to achieve further model compression. Wei et al. (2021) obtained a deep convolutional neural network model with sparse parameters by training the convolutional neural network model with sparse regularization, and combined the sparsity of the convolutional and batch regression layers to perform structured pruning to remove redundant filters. Ziani et al. (2018) proposed a vertical partition pruning method based on the maximum frequent item set, which effectively prunes the potential search space to search for optimal solutions. Zhang H. et al. (2020) performed model compression by enforcing channel-level sparsity pruning in a YOLOv3 network, and tested the effect of different gradient optimizers on model pruning before finally using the Adam optimizer to optimize the model. Jia et al. (2021) proposed a novel solution for minutely significant target object detection, which evaluates the parameters in the training model based on significant energy levels as a way to distinguish between background parameters in the model as a way to distinguish between background and salient objects.

The above-mentioned deep learning-based model compression methods still have problems such as requiring large hardware resources for acceleration, high redundancy, the stability and robustness of the network after model compression is difficult to be guaranteed in complex environments, and the network model has insufficient self-adaptability.

## 3 Improved Geometric Median Filter Based Pruning Algorithm

### 3.1 Model Pruning Methods

**FIGURE 1:**
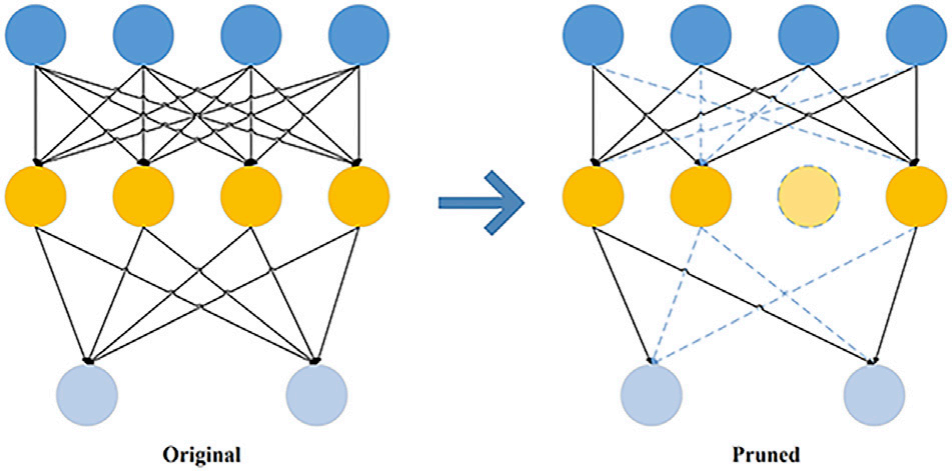
Unstructured pruning diagram.

There are two main types of model pruning methods: unstructured pruning and structured pruning. Unstructured pruning prunes the neuron or connection weights, which means that some non-0 elements in the network calculation are set to 0, or the dense connections of the network are turned into sparse connections, turning the original dense matrix operation into a sparse matrix operation, as shown in Figure 1. In Figure 1, the dashed box is a pruning of the neurons to 0, and the dashed connection is a pruning of the dense connections to sparse connections, i.e. pruning weights.

**FIGURE 2:**
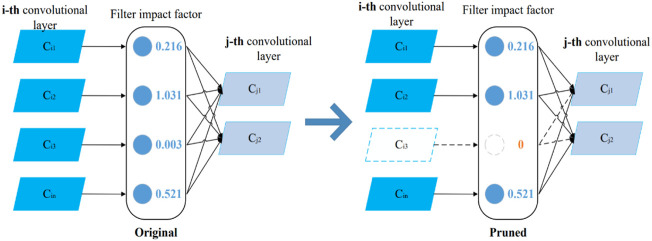
Structured pruning diagram.

Structured pruning is a type of pruning at the filter level, which focuses on pruning the filters with smaller contributions in each layer of the network. When the filter van value (the filter's impact factor) is less than a set range, the network is structured to prune redundant filters according to the van value, as shown in Figure 2. In the figure, the jth convolutional layer is the i + 1th convolutional layer. Thus, structured pruning can effectively reduce the network model size without destroying the convolutional structure.

Since the convolution kernel obtained after pruning is sparse, and most GPUs today do not provide additional acceleration for sparse matrix operations, this results in a pruned network that is not accelerated in any way compared to the original network, but may be slower.

Therefore, structured pruning is now a more general approach, and is relatively more efficient than unstructured pruning methods. For the use of the pruned network does not require the support of specific hardware platforms, computational libraries, effectively avoiding the drawbacks of unstructured pruning and enabling direct deployment on the mainstream deep learning frameworks nowadays (Liu et al., 2017).

### 3.2 Geometric Median Filtering Based Detection Model Pruning Algorithm

Model-structured pruning requires a criterion to select the filter to be pruned, i.e. the filter’s magnitude value. The most common pruning criterion is that the filter's parametric value is compared to some threshold value and if it is below the threshold, the filter is set to zero, i.e., the filter is pruned and pruned.

He et al. (2019) proposed a new filter pruning method for pruning models by geometric median filter pruning, which is a type of structured pruning.

Unlike the previous methods, geometric median filter-based pruning compresses the convolutional neural network model by removing redundant filters. Geometric median filtering works by calculating the geometric median of the filters within the same layer and, depending on the properties of the geometric median, filters near the geometric median can be represented by the remaining filters. Therefore, pruning the geometric median filter does not have a substantial negative impact on the model performance.

In d-dimensional space, given any set of n points: $a^{\left(1\right)},..,a^{\left(n\right)}$ , and $a^{\left(\text{i}\right)}\in R^{d}$ , there exists a point $x^{*}$ such that the sum of the Euclidean distances (Euclidean distances) to each point is minimized, and the point $x^{*}$ is referred to as the Geometric Median (GM) point and is calculated as:

$$x^{*}=\underset{x\in R^{d}}{\arg\min}f\left(x\right)$$ (1)

$$f\left(x\right)\overset{def}{=}\sum_{i\in \left[1,n\right]}{\left\|x-a^{\left(i\right)}\right\|}_{2}$$ (2)

In which,

$x^{*}\in R^{d}$ , and $x^{*}$ is referred to as the geometric median point;

argmin denotes the value of the variable at which the objective function f(x) is made to take its minimum valu;

[1, n] = {1, …, n}; def means that the f(x) function is defined as $\sum_{i\in \left[1,n\right]}{\left\|x-a^{\left(i\right)}\right\|}_{2}$ .

The geometric median is a classical robust estimator of data centeredness in Euclidean space and is used when pruning the model to obtain common information about all filters within a single layer i as the geometric median for that layer $F_{i}^{GM}$ .

$$F_{i}^{GM}=\underset{x\in R^{N_{i}\times H_{i}\times W_{i}}}{\arg\min}g\left(x\right)$$ (3)

$$g\left(x\right)\overset{def}{=}\sum_{j^{\prime }\in \left[1,N_{i+1}\right]}{\left\|x-F_{i,j^{\prime }}\right\|}_{2}$$ (4)

In which, g(x) denotes the sum of the Euclidean distances of all filters within tensor x to layer i. $x\in R^{N_{i}\times H_{i}\times W_{i}}$ denotes that x exists within input tensor $N_{i}\times H_{i}\times W_{i}$ , and $N_{i}$ , $H_{i}$ and $W_{i}$ denote the number of channels, height and width of the input tensor within layer i, respectively. $N_{i+1}$ indicates that the output is $N_{i+1}$ when the input is $N_{i}$ .

The core idea of geometric median filtering is that if there are filters within layer i that are close to geometric median $F_{i}^{GM}$ , then these filters are redundant and clipping these redundant filters will not have a large impact on network performance. In layer i, these redundant filters are:

$$F_{i,j^{*}}=\underset{j^{\prime }\in \left[1,N_{i+1}\right]}{\arg\min}{\left\|F_{i,j^{\prime }}-F_{i}^{GM}\right\|}_{2}$$ (5)

And these redundant filters are close to the geometric median $F_{i}^{GM}$ .

$${\left\|F_{i,j^{*}}-F_{i}^{GM}\right\|}_{2}=0$$ (6)

That is, Eq. 5 is equivalent to

$$\begin{array}{l} F_{i,j^{*}}∼\underset{j^{\prime },j^{*}\in \left[1,N_{i+1}\right]}{\arg\min}{\left\|F_{i,j^{\prime }}-F_{i,j^{*}}\right\|}_{2}\\ \;\;\;\;\;\;=\underset{j^{\prime },j^{*}\in \left[1,N_{i+1}\right]}{\arg\min}{\left\|x-F_{i,j^{\prime }}\right\|}_{2}\\ \;\;\;\;\;\;=\underset{j^{\prime },j^{*}\in \left[1,N_{i+1}\right]}{\arg\min}g\left(x\right) \end{array}$$ (7)

In Eq. 7, $x\in \left\{F_{i,1},...,F_{i,N_{i+1}}\right\}$ 。

The geometric median is a classical robust estimator of data-centricity in Euclidean space. This shows that the information of the selected filter $F_{i,j^{*}}$ can be replaced by other filters. After fine-tuning, the network can easily recover its original performance. Therefore, the neural network is pruned to have little impact on the final result of the detection.

**FIGURE 3:**
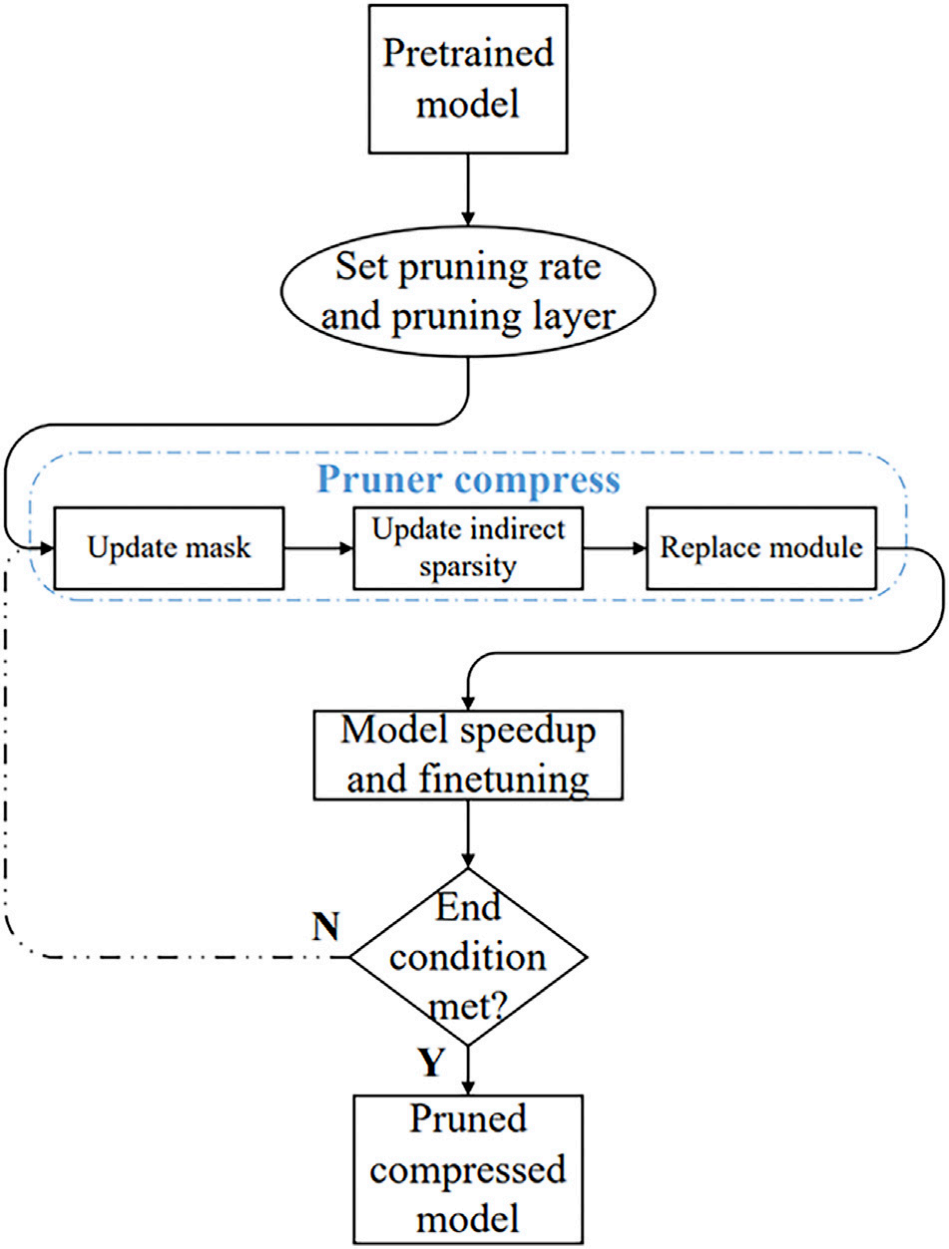
Structured pruning flow chart.

The pruning flow chart based on geometric median structured pruning is shown in Figure 3. First, a pre-trained detection model with the required compression is input and the pruning rate and pruning layers are set. The pruning rate can be set to 0–1 and the pruning layers can be set to convolutional layers, fully connected layers, Batchnorm layers, etc.

The structured pruning process in this paper includes updating the mask, updating the indirect sparsity and updating the module. After the pruning process, the model is accelerated and refined to optimize the model. Finally, an evaluation score is calculated to determine whether the end condition is met. If the end condition is met, the pruned and compressed model is output; if not, the pruning process continues.

Geometric median filtering algorithms can effectively improve the compression rate of neural networks and reduce detection model redundancy. The pruned detection model can be deployed to portable devices for faster processing (Ran et al., 2022).

In this paper, a model pruning algorithm based on geometric median filtering is used to compress the steel plate surface defect detection network and implement a model pruning defect segmentation detection algorithm based on geometric median filtering to reduce the number of parameters and computational effort of the detection model.

## 4 Experimental Results and Analysis

### 4.1 Open Source Surface Defect Dataset for Steel Plates

The Severstal dataset was released open source on the competition platform Kaggle. The Severstal dataset contains 12,568 images from the training set and 1,801 images from the test set. There are 5,902 defect-free images and 6,666 defective images in the training set. The number of defective and non-defective images in the dataset is roughly equal, and most of the images have no defects or contain only one type of defect (Hao et al., 2022).

**FIGURE 4:**
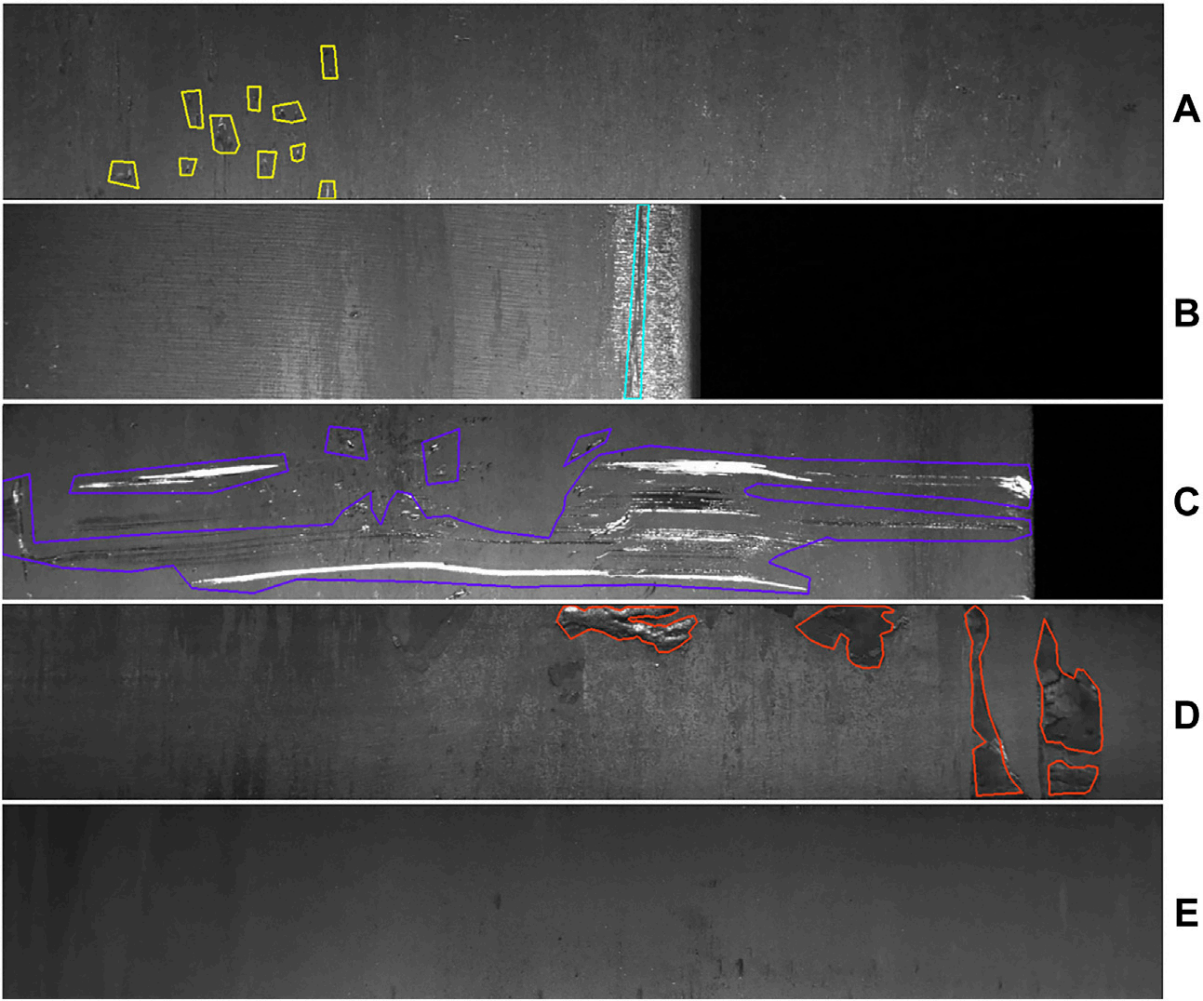
Surface defect data for Severstal plates. **(A)** Pit defects, **(B)** Edge crack defects, **(C)** Scratch and scrape defects, **(D)** Rolled-in scale defects and **(E)** Non-defect images.

All images in the Severstal dataset have a vertical and horizontal resolution of 256 and 1,600 respectively. There are four types of steel surface defects in the Severstal dataset, as shown in Figure 4: A) Pit defects, B) Edge crack defects, C) Scratch and scrape defects, D) Rolled-in scale defects and E) Non-defect images.

The Severstal dataset contains a large variation in morphology between different defects on the surface of steel plates, both large defects such as scratches and scrapes, and very small defects such as pits and edge cracks.

The extremely large span of defect scales places high demands on the defect detection segmentation algorithm: it has to focus on the details to achieve fine segmentation; and it has to focus on the global picture and have sufficient sensory field for large scale defects. These factors make feature extraction and detection segmentation of the network difficult and lead to the need for pruning and compression of the defect detection model.

### 4.2 Experimental Environment Configuration

The algorithm research and network training in this paper were conducted on a laboratory server. The specific computer systems used and the configuration of the experimental environment are shown in Table 2.

**TABLE 2:** Experimental environment configuration.

Project	Configuration
Operating system	Windows10
CPU	i7-9700k
GPU	RTX2080 Ti
RAM	DDR5 16GB × 4
Programming language	Python3.7
Deep learning framework	PyTorch1.10

This paper uses relevant open source libraries and toolkits to implement the overall algorithmic procedure based on the good ecology and scalability of the Python language and the open source framework PyTorch (Bai et al., 2021).

These open source tools greatly save the development time of the defect detection and segmentation procedure in this paper, thus allowing more time and effort to be devoted to the research, improvement and experimentation of the structured pruning algorithm.

### 4.3 Experiments on Surface Defect Segmentation Detection of Steel Plates Based on Structured Pruning

In order to verify the practical effectiveness of the proposed defect segmentation algorithm based on geometric median filter pruning, experiments with different pruning rates were conducted on different models under the same conditions to test the effect of different pruning rates on the accuracy of the models.

Since the main network layer of the pruned model is the convolutional layer, this paper only detects pruning on the convolutional layer of the detection model, and does not perform pruning experiments on the fully connected layers, Batchnorm layers, etc.

The input size of the model only affects the computational volume of the model and does not affect the number of model parameters. Therefore, the input size was set to [3, 64, 64] for the model pruning experiments, i.e., the simulated input image size was 64 × 64 for the 3-channel image.

The ResNet50 model has good performance in image recognition and localization tasks (He et al., 2016). The ResNeXt50 model is a grouped convolution based on the ResNet50 model, which can greatly reduce the number of parameters and is more effective in many visual recognition tasks (Xie et al., 2017).

The FPN-ResNeSt50 model is an improved fusion of the FPN (Feature pyramid networks) and the ResNeSt50 model (Lin et al., 2017; Zhang Y. et al., 2020), with powerful feature extraction and fusion capabilities, and have good detection capability for defect segmentation detection tasks on steel plate surfaces.

In this paper, ResNet50, ResNeXt50 and FPN-ResNeSt50 are used as the detection models for defects on the surface of steel plates, and pruning experiments and validation are performed on them.

The effect of different pruning rates on the ResNet50 model is shown in Table 3. A pruning rate of 0% indicates that no pruning is applied to the model. For example, when the pruning rate is 40%, the computation of the model is $171.84\times 10^{6}$ , which is 48.81% lower than the computation of the original model, and the number of parameters of the model is $13.15\times 10^{6}$ , which is 48.43% lower than the number of parameters of the original model.

**TABLE 3:** Effect of different pruning rates on the ResNet50 model.

Pruning rate/%	Calculated volume/M	Number of parameters/M	Calculated volume decline rate/%	Rate of decline in number of parameters/%
0	335.69	25.50	0	0
10	291.82	22.17	13.07	13.06
20	249.37	18.98	25.71	25.57
30	208.96	15.98	37.75	37.33
40	171.84	13.15	48.81	48.43
50	136.86	10.50	59.23	58.82
60	105.67	8.08	68.52	68.31
70	74.70	5.72	77.75	77.57
80	42.31	3.38	87.40	86.75
90	13.79	1.29	95.89	94.94

The effect of different pruning rates on the ResNeXt50 (32 × 4d) model is shown in Table 4. As the pruning rate increases, the computational volume and number of parameters of the network decreases and the rate of decrease in computational volume and number of parameters increases.

**TABLE 4:** Effect of different pruning rates on the ResNeXt50 (32 × 4d) model.

Pruning rate/%	Calculated volume/M	Number of parameters/M	Calculated volume decline rate/%	Rate of decline in number of parameters/%
0	347.23	24.96	0	0
10	342.73	24.75	1.3	0.84
20	336.55	24.13	3.08	3.25
30	328.34	23.21	5.44	7.01
40	310.92	21.73	10.43	12.94
50	288.07	19.81	17.04	20.63
60	251.99	16.96	27.43	32.05
70	183.47	12.63	47.16	49.40
80	112.31	8.07	67.66	67.67
90	37.06	3.04	89.33	87.82

However, the structured pruning effect of the model was not evident at smaller pruning rates in the early stages due to the ResNeXt50 (32 × 4d) model having a 32-component group convolution, resulting in a smaller pruning rate.

The effects of different pruning rates on the FPN-ResNeSt50 model are shown in Table 5.

**TABLE 5:** Effect of different pruning rates on the FPN-ResNeSt50 model.

Pruning rate/%	Calculated volume/M	Number of parameters/M	Calculated volume decline rate/%	Rate of decline in number of parameters/%
0	508.19	27.98	0	0
10	464.23	25.94	8.65	7.28
20	425.51	23.25	16.27	16.91
30	372.55	20.01	26.69	28.49
40	325.04	17.56	36.04	37.23
50	268.02	14.34	47.26	48.74
60	212.93	11.31	58.10	59.59
70	152.86	8.18	69.92	70.78
80	95.13	5.24	81.28	81.29
90	31.46	2.14	93.81	92.36

Comparing Tables 3, 4, 5, the results of the pruning experiments prove that the more grouped convolutions a network model has, the lower the compression rate of its pruning. Since grouped convolutions can greatly reduce the number of model parameters, the more groupings exist for grouped convolutions, the lower the pruning compression rate.

**FIGURE 5:**
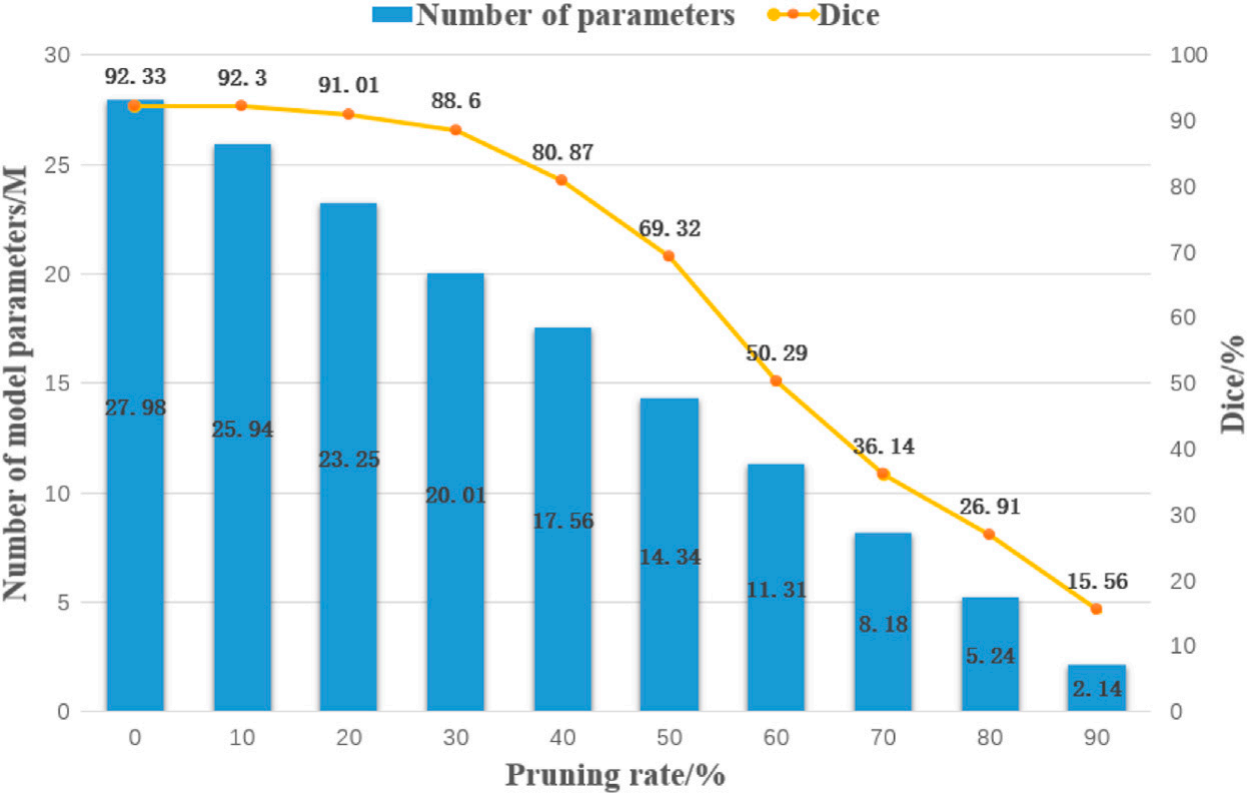
Plot of test results for different pruning rates.

The model pruning algorithm based on geometric median filtering prunes and compresses the steel plate surface defect segmentation model based on depth feature fusion, and experiments with different pruning rates were conducted on it under the same conditions to test the effect of different pruning rates on the accuracy of the FPN-ResNeSt50 model, and the detection results are shown in Figure 5.

At a pruning rate of 40%, the defect detection accuracy starts to gradually decline, so at a pruning rate greater than 40%, it will lead to the loss of important parameters of the model, resulting in a serious decline in accuracy. In contrast, at a pruning rate of 10%–30%, the model accuracy is able to maintain a low loss of accuracy.

The test results show that when the pruning rate is small, pruning brings regularization to the network and enhances the generalization performance of the network; when the pruning rate is large, the characterization ability of the network is severely damaged and the performance of the model decreases significantly.

## 5 Conclusion

In order to solve the problems of large number of model parameters and difficulty in applying the model to actual plant equipment, this paper investigates the defect segmentation detection algorithm based on geometric median filter pruning. Based on the structured pruning, a model pruning algorithm based on geometric median filtering is proposed to prune and compress the defect segmentation detection network, which greatly reduces the network parameters and computational effort and improves the generalization ability of the model. Through experimental comparisons and optimizations, the detection accuracy of steel surface defects is improved. Meanwhile, the parameters and computation of the detection model are reduced. The pruning and compression algorithm proposed in this paper has good prospects for application in the segmentation and detection of defects on steel plate surfaces. Good pruning algorithms can be applied to a variety of factory embedded or portable mobile devices and can meet the demand for real-time scene detection. In the future, there is still a long way to go in model pruning and compression research.

## Data Availability

The original contributions presented in the study are included in the article/Supplementary Material, further inquiries can be directed to the corresponding authors.
